# Angulation Between the Occipital Condyle and the Hypoglossal Canal: An Anatomical Study With Application in Transcondylar Approaches and Occipital Condyle Screw Placement

**DOI:** 10.7759/cureus.32326

**Published:** 2022-12-08

**Authors:** Humza Faisal Siddiqui, Juan J Cardona, Arada Chaiyamoon, Aishwarya Gilkes, Mathangi Rajaram-Gilkes, Sassan Keshavarzi, Joe Iwanaga, Aaron S Dumont, R. Shane Tubbs

**Affiliations:** 1 Department of Neurosurgery, Jinnah Postgraduate Medical Centre, Karachi, PAK; 2 Department of Neurosurgery, Tulane University School of Medicine, New Orleans, USA; 3 Department of Anatomy, Faculty of Medicine, Khon Kaen University, Khon Kaen, THA; 4 Department of Anatomical Sciences, St. George’s University, St. George’s, GRD; 5 Department of Medical Education, Geisinger Commonwealth School of Medicine, Scranton, USA; 6 Department of Neurology, Tulane University School of Medicine, Tulane Center for Clinical Neurosciences, New Orleans, USA; 7 Department of Structural & Cellular Biology, Tulane University School of Medicine, New Orleans, USA; 8 Department of Surgery, Tulane University School of Medicine, New Orleans, USA; 9 Department of Neurosurgery and Ochsner Neuroscience Institute, Ochsner Health System, New Orleans, USA

**Keywords:** anatomical study, transcondylar approach, craniovertebral junction, hypoglossal canal, occipital condyles

## Abstract

Background

A detailed understanding of the relationship between the occipital condyle (OC) and the deeper-lying hypoglossal canal (HC) is necessary for surgeons who place screws into the OC or drill through or around the HC. Therefore, this anatomical study was performed.

Methodology

A total of 30 skulls (60 sides) underwent an analysis of the angle formed between the long axis of the OC and the HC, i.e., the OC/HC angle. Additionally, the lengths and widths of the OCs and foramen magnum (FM) of each skull were measured using a micrometer. Statistical analyses were performed between the left and right sides, and a Pearson’s correlation coefficient was calculated between OC/HC angles and the sizes of the OCs and FM of the skulls.

Results

The OC/HC angle for the left and right sides ranged from 30 to 56 degrees (mean 46 degrees). The width of the OCs ranged from 9 to 18 mm (mean 13 mm). The length of the OCs was 18 to 31 mm (mean 24 mm). The mean length and width of the FM were 36 mm and 30 mm, respectively. There was no statistically significant difference between the OC/HC angle comparing left and right sides or male or female specimens. Additionally, no statistically significant differences were found between septated and non-septated HC. Pearson’s correlation coefficient for left and right OC/HC angles and left and right OC lengths was r = 0.4056 and r = 0.2378, respectively. Pearson’s correlation coefficient for left and right OC/HC angles and left and right OC width was r = 0.3035 and r = 0.3530, respectively. Pearson’s correlation coefficient for left and right OC/HC angles and the width of the FM was r = 0.2178 and r = 0.2048, respectively. Pearson’s correlation coefficient for left and right OC/HC angles and the length of the FM was r = 0.3319 and r = 0.2683, respectively.

Conclusions

The OC/HC angle as measured here was relatively consistent with no statistically significant differences between sides. We did not find a strong correlation between the width or length of the OC or the width or length of the FM and the OC/HC angles. Therefore, based on our study, surgeons can expect that this angle will range between 30 and 56 degrees (mean 46 degrees). Such knowledge might decrease patient morbidity following invasive procedures involving the OC.

## Introduction

The occipital condyles (OCs) are distinctive bony structures that lie laterally on either side of the foramen magnum (FM) and articulate with the superior facets of the atlas. The stability of the craniovertebral junction (CVJ) depends on the anatomical parameters of the OC and the integrity of the regional ligaments which enable the skull to be extended and flexed. Each condyle is oriented obliquely so that its anterior end is proximal to the midline [1,2]. OCs are located inferior and anterolaterally to the internal opening of the hypoglossal canal (HC), medial to jugular foramen, and inferomedial to the condylar canal. Several nerves emerging from these foramina are in the region surrounding the OC; for example, cranial nerves IX-XII, C1 and C2 spinal nerves. In addition, the caudal end of the medulla oblongata, the rostral end of the spinal cord, the inferior vermis, and the tonsils of the cerebellum are located in proximity to the OC. Vessels such as the vertebral, cerebellar, meningeal arteries, dural venous sinuses, and internal jugular veins are also closely related to it [3,4].

Extradural and intradural tumors and vascular malformations such as meningioma, schwannoma, brainstem gliomas, subarachnoid cysts, and vertebral artery aneurysms are frequently encountered in the cranial base near the FM. Deeply located lesions present a clinical challenge during craniovertebral surgeries owing to their complex anatomy. Nowadays, the dorsolateral surgical approach is preferred at the CVJ because the route is shorter and more direct, and the ventral approach entails an unacceptably higher rate of morbidity [4-7].

The basic far-lateral exposure provides access for the following three approaches: the transcondylar approach directed through the OC or the atlantooccipital joint provides access to the lower clivus and pre-medullary area; the supracondylar approach provides access to the area medial to the HC and jugular tubercle; and the paracondylar approach gives access to the posterior part of the jugular foramen, as well as the posterior aspect of the facial nerve and mastoid on the lateral side of that foramen. These approaches also make brainstem retraction minimal [8,9].

The HC extends from the posterior cranial fossa to the external skull base and is located above the OC at the approximate junction of its anterior one-third and posterior two-thirds. The hypoglossal nerve travels along with the meningeal branch of the ascending pharyngeal artery and the emissary vein from the basilar plexus through the HC [10,11]. To minimize morbidities during transcondylar, supracondylar, and paracondylar surgical approaches, it is very important to understand the anatomical relationship between the OC and HC. Therefore, our aim in this study is to determine the precise angle between the OC and HC, delineate its anatomical variations to provide surgeons deeper knowledge of this morphometric information, and enhance the surgical outcomes while using the aforementioned surgical approaches and thereby enhance the outcomes. As previous studies have measured the angulation between the HC and the midline, our study chose to better specify such angulation in regard to the ipsilateral OC for improved surgical precision.

## Materials and methods

**Figure 1:**
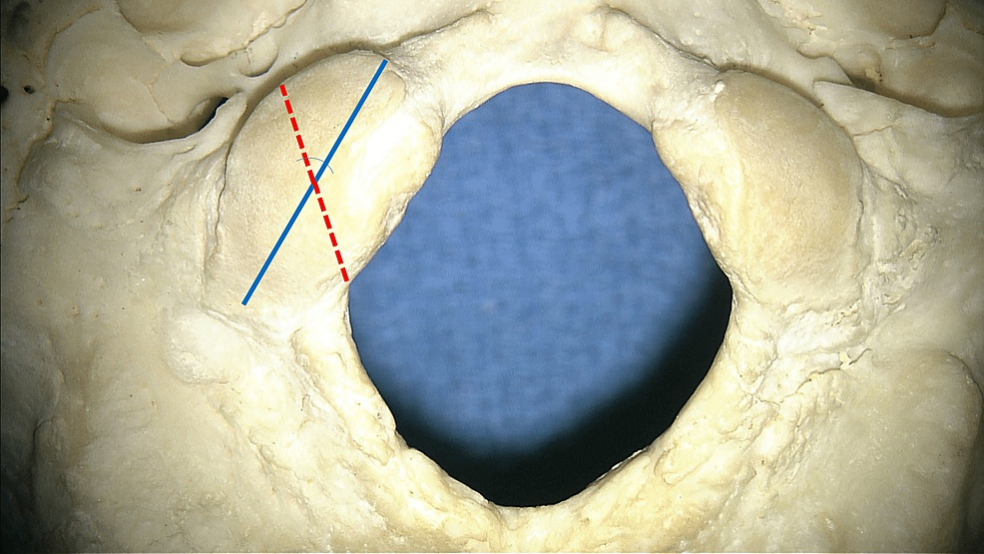
Skull specimen, seen from below, noting the occipital condyle/hypoglossal canal angle made in this study for the right occipital condyle and hypoglossal canal. The blue line is along the long axis of the occipital condyle, and the red dotted line represents the underlying hypoglossal canal.

A total of 30 adult skulls (60 sides) underwent an analysis of the angle formed between the long axis of the OC and the HC, i.e., the OC/HC angle (Figure 1). After inserting a metal wire through the HC, the angle between this line and the long axis of the OC (Figure 1) was studied. All angles were calculated using a goniometer. In total, 14 skulls were from males and 16 from females. The skull specimens were derived from the skeletal teaching collection at Tulane University School of Medicine. These were derived primarily from North American adult cadavers whose exact ages were not known. Additionally, the lengths and widths of the OCs and FM of each skull were measured using a micrometer (Mitutoyo, Japan). Each measurement was made three times and the average was taken by a single observer (RST). Statistical analyses were performed between the left and right sides (significance set at p < 0.05), and a Pearson’s correlation coefficient was calculated between the OC/HC angles and the sizes of the OCs and FM of the skulls. Every effort was made to follow all local and international ethical guidelines and laws that pertain to the use of human cadaveric donors in anatomical research [12]. Tulane University Insitutional Review Board does not require approval of non-patient/living human studies. Thus, as our study used cadavers, approval was not required.

## Results

**Figure 2:**
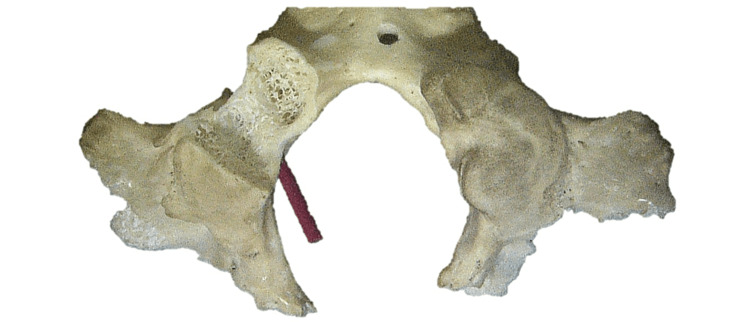
Skull specimen where the right occipital condyle has been drilled to demonstrate the more deeply positioned hypoglossal canal, here with a red vessel loop running through it. Notice the surrounding spongy bone compared to the denser cortical bone forming the hypoglossal canal.

The OC/HC angle for the left and right sides ranged from 30 to 56 degrees (mean 46 degrees). For the left sides, this angle ranged from 30 to 56 degrees (mean 47 degrees), and for the right sides, this angle ranged from 39 to 56 degrees (mean 45 degrees) (Figure 2).

A septated HC was identified on two left (3.3%) and four right sides (6.7%). The width of the OCs ranged from 9 to 18 mm (mean 13 mm). The width for the left sides ranged from 10 to 17 mm (mean 13.3 mm). The width for the right sides ranged from 9 to 18 mm (mean 13 mm). The length of the OCs was 18 to 31 mm (mean 24 mm). The length of the left sides ranged from 18 to 27 mm (mean 23 mm). The length of the right sides ranged from 19 to 31 mm (mean 24 mm). The mean length and width of the FM was 36 mm (range 31 mm to 46 mm) and 30 mm (range 24 mm to 32 mm), respectively (Table 1). No significant elevations in the HC in the sagittal plane were observed.

**Table 1:** Summary of morphometric measurements performed in this study. HC = hypoglossal canal; OC = occipital condyle; FM = foramen magnum

Variables		Skulls (60 sides)		P-value
Sex	Males	14 (28 sides)		>0.05
	Females	16 (32 sides)		>0.05
Septated HC	Left	2 sides (3.3%)		>0.05
	Right	4 sides (6.7%)		>0.05
	Side	Range	mean	
OC/HC angle degrees	Left	30–56	47	>0.05
	Right	39–56	45	>0.05
	Left and right	30–56	46	
Width	OCs	9–18 mm	13 mm	
	Left OCs	10–17mm	13.3 mm	
	Right OCs	9–18 mm	13 mm	
Length	OCs	18–31 mm	24 mm	
	Left OCs	18–27 mm	23 mm	
	Right OCs	19–31 mm	24 mm	
Pearson’s correlation coefficient			r	
Left OC/HC angle and left OC length			0.4056	
Right OC/HC angle and left OC length			0.2378	
Left OC/HC angle and left OC width			0.3035	
Right OC/HC angle and left OC width			0.353	
Left OC/HC angle and FM width			0.2178	
Right OC/HC angle and FM width			0.2048	
Left OC/HC angle and FM length			0.3319	
Right OC/HC angle and FM length			0.2683	

There was no statistically significant difference between the OC/HC angle comparing left and right sides or male or female specimens (p > 0.05). Additionally, no statistically significant differences were found between septated versus non-septated HC (p > 0.05). Pearson’s correlation coefficient for left and right OC/HC angles and left and right OC lengths was r = 0.4056 and r = 0.2378, respectively. Pearson’s correlation coefficient for left and right OC/HC angles and left and right OC width was r = 0.3035 and r = 0.3530, respectively. Pearson’s correlation coefficient for left and right OC/HC angles and the width of the FM was r = 0.2178 and r = 0.2048, respectively. Pearson’s correlation coefficient for left and right OC/HC angles and the length of the FM was r = 0.3319 and r = 0.2683, respectively (Table 1).

## Discussion

Posterior surgical approaches of the atlantooccipital joint (transcondylar, supracondylar, and paracondylar) can achieve greater stability of the joint, less morbidity and mortality, and more nearly optimal tumor resection than anterior approaches to this region. The key step during these procedures is drilling at and around the OC, which risks entry into the HC, leading to iatrogenic injury to the neurovascular structures traversing it [3,4,6,8]. However, there is little information in the literature about the precise angle between the HC and OC and the potential injury to cranial nerve XII regarding this relationship. Therefore, knowledge of the topographic relationship between the OC and HC and morphometric variations of these structures among individuals is crucial for neurosurgeons to ensure an adequate approach and safe exposure of vital anatomical structures.

Variability among the morphological parameters of the OC such as length, thickness, and condylar angle is surgically significant because it affects lateral surgical approaches. The length of the OC reflects potential craniovertebral instability after partial condylectomy; a shorter OC leads to greater instability, and a longer one can necessitate extensive resection to achieve better visualization. Similarly, the thickness of the OC determines the deepest point during vertical drilling of the condyle and the potential safety of surrounding structures, specifically the HC. A wider sagittal condylar angle is beneficial for reaching the anterior FM. Lyrtzis et al. [13] reported equal proportions of short and long OCs, 27.7% and 26.2%, respectively, among the Greek population; the mean length was 23.69 mm. However, Naderi et al. [1] presented slightly different findings. The OC was short in 35 (8.6%) cases, moderate in 312 (77.2%), and long in 57 (14.1%) among the Turkish population, with the right side being longer than the left. The mean condylar length was 23.4 mm, the depth was 9.2 mm, and the sagittal intercondylar angle was 59.3 degrees. Thanduri et al. [14] also found the right side to be longer than the left in an Indian population. Verma et al. [4] reported short OCs in 13% of skulls and long OCs in 25% among North Indians, higher than previous studies. The mean length and thickness of the OC were 23.22 ± 2.3 mm and 11.22 ± 1.4 mm, respectively.

The shape of the OC is also important in establishing the extremity of condylectomy. Several variants have been described, including oval, quadrilateral, triangular, two-portioned, kidney-shaped, ring-like, and figure-of-eight. The oval type predominated among Turkish people [1]. In central India, the most common type was quadrilateral on the right side (23.5%) and triangular on the left (29.4%) [14]. Verma et al. [4] reported that the most frequent shape of the OC was oval (53% of skulls), and high-risk types (triangular, reniform, and irregular) were observed in 15% of their specimens. The oval shape favors screw insertion during fixation as it provides a larger surface area [15]. Triangular, reniform, and other deformed shapes pose substantial difficulties and require extensive resection of the condyle during surgery [16].

Rhoton [8] reported that the intracranial end of the HC lies approximately 5 mm above the junction of the posterior and middle thirds of the OC and the extracranial end lies directly above the junction of the anterior and middle thirds of the OC. He proposed that for better anterior and superior exposure, the maximum amount of the upper OC that can be drilled without accessing the HC is the posterior third of its long axis. However, he stated that substantial drilling around the HC can translocate the hypoglossal nerve within it. Moreover, bleeding when the upper posterior portion of the OC is drilled can be from the posterior condylar vein, which surgeons can mistakenly interpret as bleeding from the venous plexus of the HC.

Barut et al. [17] examined 56 dry skulls and measured the distance between the intracranial edge of the HC and the anterior margin of the OC as 11.2 mm on both sides. The distance between the intracranial edge of the HC and the posterior margin of the OC was 12.5 mm on the right side and 12.6 mm on the left. They concluded that 12 mm of the OC can safely be resected without injuring the hypoglossal nerve. Kizilkanat et al. [18] found the distances from the intracranial end of the HC to the posterior, anterior, and inferior margins of the OC to be 12.3 ± 2.4 mm, 11.2 ± 1.6 mm, and 9.4 ± 1.2 mm, respectively, in Turkish-Caucasian skulls. In central Indian specimens, the intracranial orifice of the HC was predominantly at the junction of the second and third quarters of the OC or the second quarter, while the extracranial orifice was commonly at the junction of the first and second quarters of the OC, or the first quarter on the left side or the second quarter on the right [14]. Fetouh and Awadalla [19] found that the intracranial orifice of the HC was most often (65%) located against the junction of the second and third quarters or the third quarter of the OC in dry Arabian skulls, whereas the extracranial orifice was located at the junction of the first or second quarters or the second quarter in 69%. They concluded that drilling around the posterior quarter of the OC, 4.5 to 7.8 mm from the posterior end, entails the least risk to the HC as no orifice was found in this region. Farid and Fattah [20] reported that the mean distance between the intracranial end of the HC and the posterior margin of the OC was 12.66 mm on the right and 13.17 mm on the left in modern Egyptian skulls, slightly higher than those reported in other studies.

Lyrtzis et al. [13] stratified their data on Greek skulls according to sex and age. The distance between the HC and the posterior border of the OC on the left side was statistically higher in males, 8.38 mm, than in females, 7.97 mm. In addition, the HC was longer in males than females on the right, 9.14 mm and 8.63 mm, respectively. Three age groups were distinguished, namely, 20-39 years, 40-59 years, and 60-79 years. The remarkable decrease in distance between the HC and the posterior border of the OC across those three age groups indicates different safe drilling zones in them, i.e., 8.61 ± 1.41 mm in the first, 8.54 ± 1.19 mm in the second, and 7.92 ± 1.17 mm in the third. The authors deemed that a distance between the HC and OC of 8.34 mm in males and 7.99 mm in females was safe to drill without endangering nervous tissue, considerably shorter than other studies. The anterior and posterior intercondylar distances were substantially lower in males than in females. Bulsara et al. [21] assessed the anatomy of the HC using three-dimensional computed tomography prior to surgery involving the transcondylar approach to preclude injury to the hypoglossal nerve. The internal aperture of the HC was 11.0 ± 1.4 mm and the external aperture was 19.1 ± 2.4 mm from the superior surface of the condyle. The external opening was 12.4 ± 2.1 mm from the lowest point of the OC. The posterior condylar emissary vein was estimated to be 12.2 ± 3 mm from the intracranial opening of the HC. Parvindokht et al. [10] examined skulls of Iranian descent and observed significant differences in the distances between the HC and the anterior tip, posterior tip, and lower border of OCs on the left and right sides.

Certain morphological parameters of the HC are crucial during any surgery that can potentially cause neurovascular injury. Katsuta et al. [22] studied the microanatomy of the HC in Caucasian and Indian specimens and observed two bundles of the hypoglossal nerve that ran obliquely in it. The nerve bundles were situated superomedially as they entered the internal orifice and inferolaterally as they exited the external orifice. A septum divided the canal along its entire length in 1-3% of cases, and there was a septum in either the internal or external opening in 15-20% of cases. The authors proposed that a surgeon should not be too bothered if there is some bleeding at the posterolateral edge of the HC while the OC is being drilled, and the posterolateral bony wall of the canal can be safely drilled off without risking the nerve. Kumar et al. [23] found that spurs and septa were more common in the left than the right HC in Indian skulls. However, Paraskevas et al. [24] found that septa were more frequent on the right side in Greek specimens. They also measured the mean lateral length of the HC as 10.22 mm and its mean medial length as 8.93 mm, while the mean inclinations of single HCs relative to the sagittal plane were 42.3 degrees on the right and 32.4 degrees on the left. There was a complete osseous septum traversing the entire length of the HC in 1.72% of cases, and full osseous bridging in the internal or external openings in approximately 20% of cases. When a septated HC was found in our cohort, this did not affect the OC/HC angle. Lyrtzis et al. [13] found a complete septum in 23.6% of HCs and bony spurs in 12.9%. Double canals were found in 15.1% on the right and 6.8% on the left in males, and 13.2% on the right and 10.2% on the left in females. Verma et al. [4] reported 34% septate HCs, more frequent on the left. Another study revealed a higher prevalence of osseous septa on the right [25]. Muthukumar et al. [16] found the mean angle between the midline and HC was 49 degrees on the left and right sides in Indian skulls. This is compared to our findings where the OC/HC angle for the left and right sides ranged from 30 to 56 degrees (mean 46 degrees).

A clinically relevant correlation between the hypoglossal nerve and the OC can be established from reported cases of hypoglossal nerve injury following OC fracture [26,27]. In particular, the proximity of the OC and HC puts cranial nerve XII and associated vascular structures at risk, potentially leading to neurological deficits and hemorrhage [28]. Intraoperative monitoring using evoked potentials and electromyographic recordings of the tongue can interpret hypoglossal nerve function, but these modalities are not routinely used owing to postoperative swelling [3]. Detailed knowledge of the anatomical variations of the OC and HC is pivotal for neurosurgeons operating near the CVJ to prevent postoperative complications. There are vast morphological differences according to ethnicity, sex, and age among individuals. Precise preoperative detection of these variations by neuroimaging and careful evaluation can help in accurate surgical decision-making, reducing surgical morbidity.

The authors sincerely thank those who donated their bodies to science so that anatomical research could be performed. Results from such research can potentially increase mankind’s overall knowledge which can then improve patient care. Therefore, these donors and their families deserve our highest gratitude [29].

## Conclusions

The OCs lie inferior and anterolaterally to the internal opening of HC and laterally on either side of the FM conforming to the atlantooccipital joint along with the atlas. Tumors and vascular malformations are frequently encountered near or deep into the FM representing a challenge during CVJ surgeries. To counter these challenges, the far-lateral exposure allows access to the OC so that a transcondylar approach can be performed. This allows for greater visualization of the internal aspect of the FM. For such approaches, however, a detailed understanding of the relationship between the OC and the HC can help surgeons avoid hypoglossal nerve injury and can be useful for planning the OC screw placement. The OC/HC angle as measured here was relatively consistent with no statistically significant differences between sides. We did not find a strong correlation between the width or length of the OC or the width or length of the FM and the OC/HC angle. Therefore, based on our study, surgeons can expect that this angle will range between 30 and 56 degrees (mean 46 degrees).

## References

[1] Naderi S, Korman E, Citak G (2005). Morphometric analysis of human occipital condyle. Clin Neurol Neurosurg.

[2] Standring S (2016). Gray's Anatomy: The Anatomical Basis of Clinical Practice. https://www.worldcat.org/title/213447727.

[3] Boulton MR, Cusimano MD (2003). Foramen magnum meningiomas: concepts, classifications, and nuances. Neurosurg Focus.

[4] Verma R, Kumar S, Rai AM, Mansoor I, Mehra RD (2016). The anatomical perspective of human occipital condyle in relation to the hypoglossal canal, condylar canal, and jugular foramen and its surgical significance. J Craniovertebr Junction Spine.

[5] Ma L, Shrestha BK, You C, Hui XH (2015). Revisiting the far lateral approach in the treatment of lesions located at the craniocervical junction—Experiences from West China hospital, Sichuan University, Chengdu. Interdiscip Neurosurg.

[6] al-Mefty O, Borba LA, Aoki N, Angtuaco E, Pait TG (1996). The transcondylar approach to extradural nonneoplastic lesions of the craniovertebral junction. J Neurosurg.

[7] Wu Z, Zhang JT, Jia GJ (2004). [Postauricular tran-supracondylar approach removed jugular foramen and hypoglossal canal tumors]. Zhonghua Wai Ke Za Zhi.

[8] Rhoton AL Jr (2000). The far-lateral approach and its transcondylar, supracondylar, and paracondylar extensions. Neurosurgery.

[9] Karam YR, Menezes AH, Traynelis VC (2010). Posterolateral approaches to the craniovertebral junction. Neurosurgery.

[10] Parvindokht B, Reza DM, Saeid B (2015). Morphometric analysis of hypoglossal canal of the occipital bone in Iranian dry skulls. J Craniovertebr Junction Spine.

[11] Tatagiba M, Koerbel A, Roser F (2006). The midline suboccipital subtonsillar approach to the hypoglossal canal: surgical anatomy and clinical application. Acta Neurochir (Wien).

[12] Iwanaga J, Singh V, Takeda S (2022). Standardized statement for the ethical use of human cadaveric tissues in anatomy research papers: recommendations from Anatomical Journal Editors-in-Chief. Clin Anat.

[13] Lyrtzis C, Piagkou M, Gkioka A, Anastasopoulos N, Apostolidis S, Natsis K (2017). Foramen magnum, occipital condyles and hypoglossal canals morphometry: anatomical study with clinical implications. Folia Morphol (Warsz).

[14] Thanduri N, Rai N, Nair S, Bankwar V (2018). Occipital condyles and its relation with hypoglossal canal: Anatomical study in central Indian population. Indian J Clin Anat Physiol.

[15] Ozer MA, Celik S, Govsa F, Ulusoy MO (2011). Anatomical determination of a safe entry point for occipital condyle screw using three-dimensional landmarks. Eur Spine J.

[16] Muthukumar N, Swaminathan R, Venkatesh G, Bhanumathy SP (2005). A morphometric analysis of the foramen magnum region as it relates to the transcondylar approach. Acta Neurochir (Wien).

[17] Barut N, Kale A, Turan Suslu H, Ozturk A, Bozbuga M, Sahinoglu K (2009). Evaluation of the bony landmarks in transcondylar approach. Br J Neurosurg.

[18] Kizilkanat ED, Boyan N, Soames R, Oguz O (2006). Morphometry of the hypoglossal canal, occipital condyle, and foramen magnum. Neurosurg Q.

[19] Fetouh FA, Awadalla AM (2009). Morphometric analysis of the occipital condyle and its surgical implications in the transcondylar approach. Skull Base.

[20] Farid SA, Abdel Fattah IO (2018). Morphometric study of human adult occipital condyle, hypoglossal canal and foramen magnum in dry skull of modern egyptians. Int J Clin Develop Anat.

[21] Bulsara KR, Asaoka K, Aliabadi H, Kanaly C, Friedman A, Fukushima T (2008). Morphometric three-dimensional computed tomography anatomy of the hypoglossal canal. Neurosurg Rev.

[22] Katsuta T, Matsushima T, Wen HT, Rhoton AL Jr (2000). Trajectory of the hypoglossal nerve in the hypoglossal canal: significance for the transcondylar approach. Neurol Med Chir (Tokyo).

[23] Kumar S, Verma R, Rai AM, Mehra RD (2017). Morphological and morphometric analysis of hypoglossal canal in north Indian dry skulls and it's significance in cranial base surgeries. J Clin Diagn Res.

[24] Paraskevas GK, Tsitsopoulos PP, Papaziogas B, Kitsoulis P, Spanidou S, Tsitsopoulos P (2009). Osseous variations of the hypoglossal canal area. Med Sci Monit.

[25] Pereira GA, Lopes PT, Santos AM, Duarte RD, Piva L, Pozzobon A (2012). Morphometric analysis related to the transcondylar approach in dry skulls and computed tomography. Int J Morphol.

[26] Lam CH, Stratford J (1996). Bilateral hypoglossal nerve injury with occipital condylar fracture. Can J Neurol Sci.

[27] Chugh S, Kamian K, Depreitere B, Schwartz ML (2006). Occipital condyle fracture with associated hypoglossal nerve injury. Can J Neurol Sci.

[28] Wen HT, Rhoton AL Jr, Katsuta T, de Oliveira E (1997). Microsurgical anatomy of the transcondylar, supracondylar, and paracondylar extensions of the far-lateral approach. J Neurosurg.

[29] Iwanaga J, Singh V, Ohtsuka A (2021). Acknowledging the use of human cadaveric tissues in research papers: recommendations from anatomical journal editors. Clin Anat.

